# Risk factors for distant metastasis of Chondrosarcoma in the middle-aged and elderly people

**DOI:** 10.1097/MD.0000000000035562

**Published:** 2023-11-03

**Authors:** Guang-hua Deng

**Affiliations:** a Ya’an Hospital of Traditional Chinese Medicine, Ya'an, China.

**Keywords:** chondrosarcoma, metastasis, nomogram, SEER database

## Abstract

Chondrosarcoma is the second most common primary bone malignancy with the highest incidence in middle-aged and elderly people, where distant metastasis (DM) still leads to poor prognosis. The purpose of this study was to construct a nomogram for studying the diagnosis of DM in middle-aged and elderly patients with chondrosarcoma. Data on chondrosarcoma patients aged ≥ 40 years diagnosed from 2004 to 2015 were extracted from the Surveillance, Epidemiology, and End Results (SEER) database. The data were divided into a training set and an internal validation set according to a 7:3 ratio, and the training set data were screened for independent risk factors for DM in chondrosarcoma patients using univariate and multivariate logistic regression analysis. The screened independent risk factors were then used to build a nomogram. In addition, data from 144 patients with chondrosarcoma aged ≥ 40 years diagnosed in a tertiary hospital in China from 2012 to 2021 were collected as the external validation set. The results were evaluated by receiver operating characteristic curves, calibration curves, and decision curve analysis in the training set, internal validation set, and external validation set. A total of 1462 middle-aged and elderly patients with chondrosarcoma were included, and 92 (6.29%) had DM at the time of diagnosis. Independent risk factors for DM in middle-aged and elderly patients with chondrosarcoma included being married (OR: 2.119, 95% CI: 1.094–4.105), histological type of dedifferentiated chondrosarcoma (OR: 1.290, 95% CI: 1.110–1.499), high-grade tumor (OR: 1.511, 95% CI: 1.079–2.115), T3 stage (OR: 4.184, 95% CI: 1.977– 8.858), and N1 staging (OR: 5.666, 95% CI: 1.964–16.342). The area under the receiver operating characteristic curve (AUC) was 0.857, 0.820, and 0.859 in the training set, internal validation set, and external validation set, respectively. The results of the calibration curve and decision curve analysis also confirmed that the established nomogram could accurately predict DM in middle-aged and elderly patients with chondrosarcoma. Married, histological type of dedifferentiated chondrosarcoma, high-grade tumor, T3 stage, and N1 stage are independent risk factors for DM in middle-aged and elderly chondrosarcoma patients, and clinicians should see more attention.

## 1. Introduction

Chondrosarcoma is the second most common primary bone malignancy after osteosarcoma,^[1]^ accounting for 30% of primary chondrosarcomas and occurring in middle-aged and elderly people.^[2,3]^ The main treatment modality for chondrosarcoma is surgery, while the efficacy of radiotherapy and chemotherapy remains controversial. Patients with chondrosarcoma often have a good outcome after surgery. Laitinen et al^[4]^ showed that extensive surgical resection has a good prognosis for chondrosarcoma. Song et al^[5]^ study showed survival advantages associated with surgery for patients with low-grade chondrosarcoma. However, a small percentage of patients with chondrosarcoma present with distant metastases after being diagnosed.^[6]^ Distant metastasis (DM) is known to be an independent prognostic factor that adversely affects the survival of middle-aged and elderly patients with chondrosarcoma.^[7–9]^ Therefore, early identification of DM in middle-aged and elderly patients with chondrosarcoma is critical for patient prognosis, but current studies have not reported on independent risk factors for the development of DM in middle-aged and elderly chondrosarcoma.

In many cancer populations, the predictive accuracy of nomograms is higher than that of conventional staging systems. For example, colon cancer,^[10]^ pancreatic cancer,^[11]^ kidney cancer,^[12]^ and bladder cancer.^[13]^ Given that this statistical tool has better predictive power, this study aimed to find independent risk factors for middle-aged and elderly patients with chondrosarcoma by creating a nomogram.

Therefore, data from the Surveillance, Epidemiology and End Results (SEER) database of middle-aged and elderly patients diagnosed with chondrosarcoma from 2004 to 2015 were selected for this study, and a nomogram was created to study the diagnosis of DM in middle-aged and elderly patients with chondrosarcoma.

## 2. Patients and methods

### 2.1. Patients

Inclusion criteria were identified as ICD-O-3 Chondrosarcoma: 9220–9243; diagnosed between 2004 and 2015. Patient age ≥ 40 years. Exclusion criteria were: missing or unknown clinical information. Finally, 1462 patients diagnosed with chondrosarcoma were included in this study and all patients were randomized in a 7:3 ratio into a training set (70%) and an internal validation set (30%). Patients in the training set were used to construct the nomogram, and patients in the internal validation set were used to validate the nomogram. In addition, 144 patients with chondrosarcoma aged ≥ 40 years from a tertiary hospital in China were collected as the external validation set.

### 2.2. Data collection

In this study, the variables used to determine the risk factors for DM in patients with chondrosarcoma were as follows: age, sex, race, marital status, primary site, side, number, tumor size, grade, histological type, T stage, and N stage.

### 2.3. Statistical analysis

In this study, all statistical analyses were performed using SPSS 26.0 and R software (version 4.3.1), and *P* values < .05 were considered statistically significant. All patients with chondrosarcoma were randomly divided into training and validation sets in R software and the distribution of variables between the 2 sets was compared using the chi-square test or Fisher’s exact test.

Univariate logistic analysis was performed to identify risk factors associated with DM. Variables with *P* < .05 in the univariate analysis were included in the multivariate logistic regression analysis, and variables with *P* < .05 in the multivariate analysis were identified as independent risk factors for DM in middle-aged and elderly patients with chondrosarcoma. In addition, a new diagnostic nomogram based on independent risk factors was used to create a new diagnostic nomogram.

Receiver operating characteristic curves were plotted and the corresponding area under the curve was calculated, and the performance of the nomogram was evaluated using calibration curves and decision curve analysis.

## 3. Results

### 3.1. Baseline characteristics of the study population

A total of 1462 middle-aged and elderly patients with chondrosarcoma were screened for inclusion in the study, with 1026 patients assigned to the training set and 436 patients assigned to the internal validation set according to a 7:3 ratio. As shown in Table 1, gender was most commonly male, with 55.56% in the training set and 58.49% in the internal validation set. The most common race was white, with 89.08% in the training set and 88.99% in the internal validation set. The most common within marriage was married, with 65.89% in the training set and 67.89% in the internal validation set. At diagnosis, the most common number of tumors was only one lesion, with 74.17% in the training set and 72.94% in the internal validation set. Grades were usually grade I and II, accounting for 72.90% of the training set and 77.29% of the internal validation set. The most common histological type was chondrosarcoma, NOS, accounting for 74.37% of the training set and 88.99% of the internal validation set. The most common T stage was T1, accounting for 56.04% of the training set and 54.82% of the internal validation set. The most common N stage is N0, accounting for 98.25% of the training set and 98.62% of the internal validation set. Also, the base test or Fisher’s exact test was performed on both sets of data and a completely random distribution was found in both sets (Table 1).

**Table 1:** Baseline characteristics of the study population.

Factors	Training set	Validation set	*P*
	(n = 1026)	(n = 436)	
Age, yr			
40–59	535	221	.725
60–80	416	186	
≥80	75	29	
Sex			
Female	456	181	.301
Male	570	255	
Race			
White	914	388	.993
Black	57	24	
Other	55	24	
Marital			
Yes	676	296	.458
No	350	140	
Primary site			
Axial	449	181	.702
Limb	489	218	
Other	88	37	
Laterality			
Left	396	188	.252
Right	400	154	
Other	230	94	
Tumor number			
Only one	761	318	.623
Other	265	118	
Size, mm			
<50	310	130	.974
50–100	403	174	
>100	313	132	
Histological type			
9220	763	338	.481
9221	5	4	
9231	124	52	
9240	13	3	
9242	6	2	
9243	115	37	
Grade			
I	327	132	.135
II	421	205	
III	163	54	
IV	115	45	
T stage			
T1	575	239	.789
T2	436	192	
T3	15	5	
N stage			
N0	1008	430	.603
N1	18	6	

9220 = Chondrosarcoma, NOS, 9221 = Juxtacortical chondrosarcoma, 9231 = Myxoid chondrosarcoma, 9240 = Mesenchymal chondrosarcoma, 9242 = Clear cell chondrosarcoma, 9243 = Dedifferentiated chondrosarcoma.

### 3.2. Incidence and risk factors of DM in patients with chondrosarcoma

A total of 92 (6.29%) of 1462 middle-aged and elderly patients with chondrosarcoma had DM, and 1370 (93.71%) had no DM. As shown in Table 2, the 12 potential factors included were subjected to univariate logistic regression analysis, which revealed age, marital status, tumor size, histological type, grade, T stage, and N stage as variables associated with DM. Screening variables of interest were included in multivariate logistic regression analysis, which revealed that being married, histological type of dedifferentiated chondrosarcoma, high-grade tumor, T3 stage and N1 stage were independent risk factors for chondrosarcoma DM (Table 2).

**Table 2:** Univariate and multivariate logistic regression analysis in the training cohort.

Factors	Univariate analysis	Multivariate analysis		
	*P*	HR	95% CI	*P*
Age	.040	1.045	0.672–1.625	.846
Sex	.131			
Race	.104			
Marital	.030	2.119	1.094–4.105	.026
Primary site	.705			
Laterality	.278			
Tumor number	.164			
Size	<.001	1.605	0.927–2.780	.091
Histological type	<.001	1.290	1.110–1.499	.001
Grade	<.001	1.511	1.079–2.115	.016
T stage	<.001	4.184	1.977–8.858	<.001
N stage	<.001	5.666	1.964–16.342	.001

### 3.3. Nomogram development and validation

**Figure 1.**
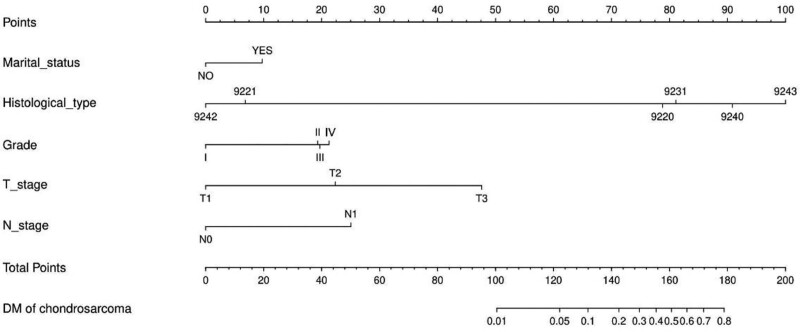
Nomogram predicting the risk of distant metastasis of chondrosarcoma middle-aged and elderly patients. 9220, Chondrosarcoma, NOS; 9221, Juxtacortical chondrosarcoma; 9231, Myxoid chondrosarcoma; 9240, Mesenchymal chondrosarcoma; 9242, Clear cell chondrosarcoma; 9243, Dedifferentiated chondrosarcoma.

**Figure 2.**
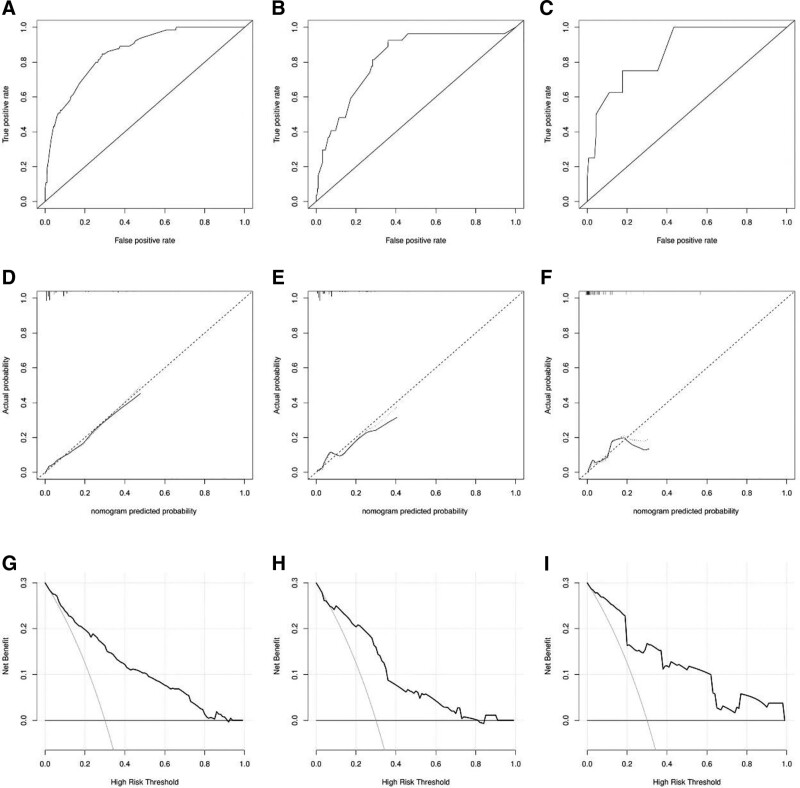
ROC curves of the Nomogram for predicting metastases in the training (A), internal validation (B), and external validation (C) sets. Calibration curves of the Nomogram for predicting metastases in the training (D), internal validation (E), and external validation (F) sets. DCA of the Nomogram for predicting metastases in the training (G), internal validation (H), and external validation (I) sets. DCA = decision curve analysis, ROC = receiver operating characteristic.

The independent risk factors screened for predicting the risk of DM due to chondrosarcoma in middle-aged and elderly patients were plotted on a nomogram (Fig. 1). Receiver operating characteristic curves were then plotted for the training set, internal validation set, and external validation set (Fig. 2A–C), and the corresponding area under the curve of 0.857, 0.820, and 0.859 were calculated. In addition, calibration curves are plotted, showing that the predicted results agree well with the observed results (Fig. 2D–F). Decision curve analysis curves were also plotted, which also showed that the predicted results were in good agreement with the observed results (Fig. 2G–I).

## 4. Discussion

Middle-aged and elderly people are people with a high incidence of chondrosarcoma have been the focus of bone oncologists. According to related studies, approximately 10% to 15% of patients with chondrosarcoma have DM at the time of diagnosis.^[6]^ Patients with chondrosarcoma with DM all have a poor prognosis, and Giuffrida et al^[7]^ found that the 30-year survival rate was more than four times higher in patients without DM than in those with DM.

There are many studies on chondrosarcoma in middle-aged and elderly people, for example, Sun et al^[14]^ found that CXCR4-targeted therapy inhibited DM of chondrosarcoma. Sheng et al^[15]^ found that SFRP5 has an important role in DM of chondrosarcoma. However, these studies are all based on the molecular level, while studies on clinical features are still relatively lacking. It is difficult for molecular-level studies to be used in the clinical setting, and for this reason, clinical characterization studies should be established.

The nomogram is a visual clinical model that combines multiple clinical characteristics and is used to predict multiple independent risk factors.^[16–19]^ The assessment of outcome indicators is accurate and concise. Therefore, there is a need to establish a nomogram on clinical features that can be used to predict DM in middle-aged and elderly patients with chondrosarcoma.

A current study^[20]^ on DM from chondrosarcoma showed that high-grade tumor, T3 stage, and large tumor size were independent risk factors for DM from chondrosarcoma, whereas being unmarried and using surgery were independent protective factors. However, the current study showed that for middle-aged and older patients with chondrosarcoma the independent risk factors for DM were married, histological type of dedifferentiated chondrosarcoma, high-grade tumor, T3 stage, and N1 stage.

Previous a study^[21]^ has confirmed the association between high-grade tumors and the T3 stage of chondrosarcoma and DM. However, a study^[22]^ has shown that tumor size is associated with DM. In the present study, this was associated with DM when the univariate analysis was performed, whereas it was not when the multifactorial analysis was performed. The covariance was due to the presence of covariance between the T stage and size, which was excluded when the multifactorial analysis was performed. Dedifferentiated chondrosarcoma is the most likely to develop DM among several chondrosarcoma subtypes. Amer et al^[23]^ also showed that patients with dedifferentiated chondrosarcoma had the worst prognosis, presumably due to the early development of DM in patients. The present study showed that married are more likely to develop DM compared to unmarried, and the present study of married included Divorced, married (including common law), separated, and widowed. Gao et al^[24]^ showed that marital status is an independent risk factor affecting the prognosis of patients with chondrosarcoma, and the prognosis of widowed patients is poor, considering that it may be related to DM. The prognosis of widowed patients is poor, so more attention should be paid to widowed patients. N1 stage was also found to be an independent risk factor in this study, and patients with lymph node infiltration were more likely to develop DM,^[25]^ which is consistent with the results of previous studies. When lymph node infiltration occurs in chondrosarcoma, it is important to be alert to the possibility of developing DM.

Although the predictive nomogram in this study showed good predictive power, there are still some limitations that need to be considered. First, to avoid interference caused by different diagnostic codes in different years, the data included were only from the clinical data of patients diagnosed with chondrosarcoma from 2004 to 2015 in the SEER database, not from all patients with chondrosarcoma since the database was established. Second, because our study is retrospective, it is inevitable that some patient data are missing. Third, although the SEER database involves different ethnic groups, the United States still consists mainly of whites and blacks, with less clinical data recorded for Asians, which may make the Nomogram somewhat limited.

## 5. Conclusion

Married, histological type of dedifferentiated chondrosarcoma, high-grade tumor, T3 stage, and N1 stage are independent risk factors for DM in middle-aged and elderly chondrosarcoma patients, and clinicians should see more attention.

## Author contributions

**Conceptualization:** Guang-hua Deng.

**Data curation:** Guang-hua Deng.

**Formal analysis:** Guang-hua Deng.

**Funding acquisition:** Guang-hua Deng.

**Investigation:** Guang-hua Deng.

**Methodology:** Guang-hua Deng.

**Project administration:** Guang-hua Deng.

**Resources:** Guang-hua Deng.

**Software:** Guang-hua Deng.

**Supervision:** Guang-hua Deng.

**Validation:** Guang-hua Deng.

**Visualization:** Guang-hua Deng.

**Writing – original draft:** Guang-hua Deng.

**Writing – review & editing:** Guang-hua Deng.
